# Synthesis of New Indole Derivatives Structurally Related to Donepezil and Their Biological Evaluation as Acetylcholinesterase Inhibitors

**DOI:** 10.3390/molecules17054811

**Published:** 2012-04-25

**Authors:** Mohamed M. Ismail, Mona M. Kamel, Lamia W. Mohamed, Samar I. Faggal

**Affiliations:** Pharmaceutical Organic Chemistry Department, Faculty of Pharmacy, Cairo University, Cairo 11562, Egypt

**Keywords:** indole, isatin, oxindole, acetylcholinesterase inhibitors, Alzheimer, donepezil

## Abstract

New series of indole derivatives analogous to donepezil, a well known anti-Alzheimer and acetylcholinesterase inhibitor drug, was synthesized. A full chemical characterization of the new compounds is provided. Biological evaluation of the new compounds as acetylcholinesterase inhibitors was performed. Most of the compounds were found to have potent acetylcholinesterase inhibitor activity compared to donepezil as standard. The compound 1-(2-(4-(2-fluorobenzyl) piperazin-1-yl)acetyl)indoline-2,3-dione **(IIId)** was found to be the most potent.

## 1. Introduction

**Figure 1:**
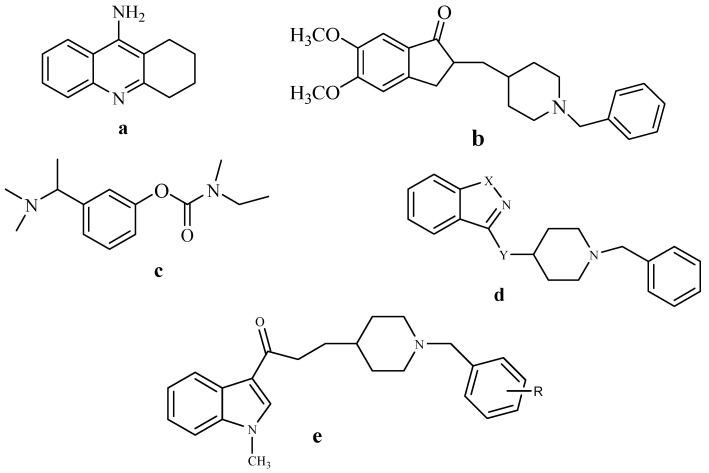
Structure of some anti-Alzheimer and acetylcholinesterase inhibitor compounds.

Among many pharmacological agents, acetylcholinesterase inhibitors (AChEI) are the only class of compounds that have consistently proven to be efficacious in treating the cognitive and functional symptoms of Alzheimer’s disease [1]. Alzheimer’s disease (AD), is one of the most severe conditions affecting elderly people. It is estimated that around 24 million people worldwide are suffering from AD. The figure is expected to increase significantly over the next 50 years due to increasing life expectancy [2]. Alzheimer’s disease is described as a degenerative disease of the central nervous system (CNS) characterized in particular by premature senile mental deterioration [3]. Many drugs were approved by FDA for the treatment of AD through inhibition of acetylcholinesterase enzyme, Tacrine (**a**, Figure 1) [4] was the first drug approved by FDA. Later, donepezil (**b**, Figure 1) [5] and rivastigmine (**c**, Figure 1) [6] were used instead of tacrine due to the associated side effects, including liver damage [4]. Several analogues of donepezil were reported as potent acetylcholinesterase inhibitors such as (**d**) [7] and (**e**) [8] (Figure 1).

The main objective of the present study was to design and synthesize novel active indole derivatives displaying anti-Alzheimer activity through inhibition of acetylcholinesterase enzyme based on structural modification of donepezil. The new derivatives were synthesized by using indole moiety as a bioisosteric substitute of the indanone moiety of donepezil and including an acetamido group as a spacer since it was reported to be important for acetylcholinesterase inhibitor activity [9]. In addition, the new derivatives were tested for their capacity to inhibit acetylcholinesterase enzyme. 

## 2. Results and Discussion

### 2.1. Chemistry

**Scheme 1:**
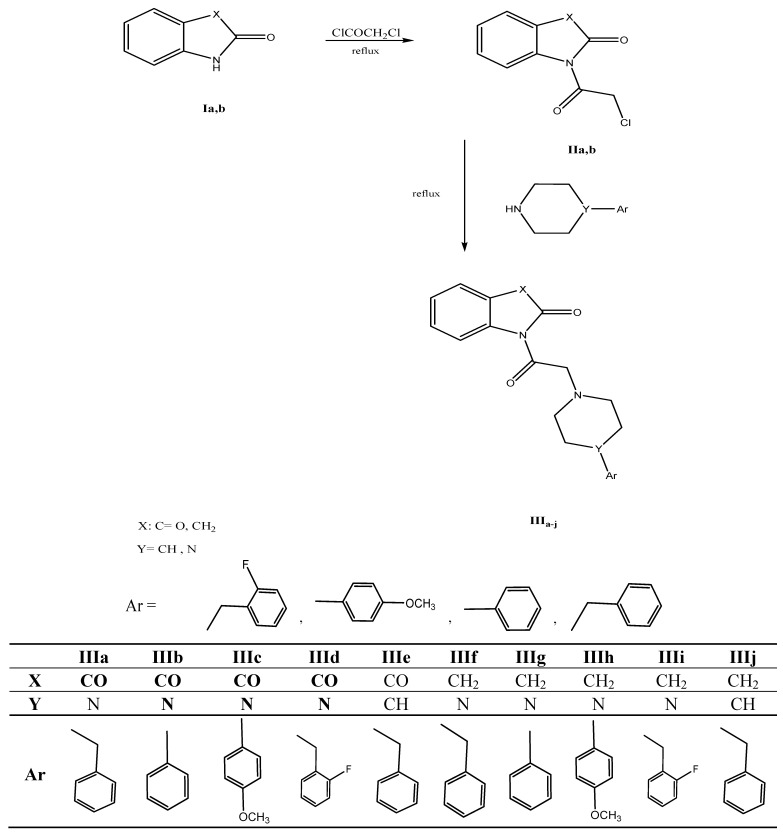
Synthesis of target compounds **IIIa**–**j**.

**Scheme 2:**
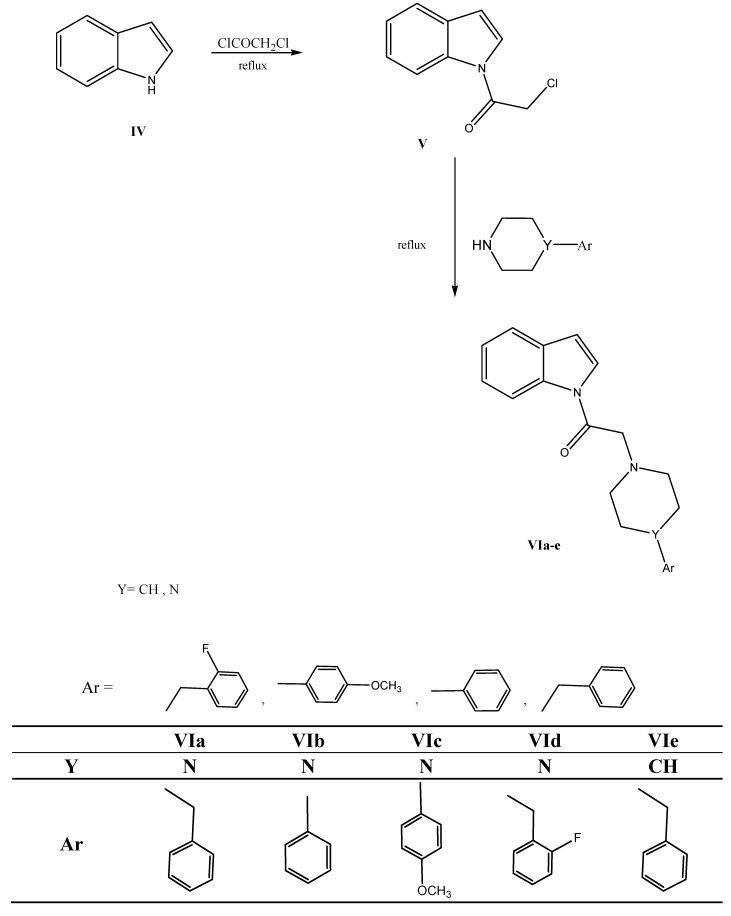
Synthesis of target compounds **VIa**–**e**.

The synthesis of the new derivatives is outlined in Scheme 1 and Scheme 2. Certain intermediates were synthesized first by chloroacetylation of indole and isatin adopting reported procedures [10,11]. Chloroacetylation of oxindole was performed in the absence of solvent producing **IIb** in fairly good yield. The structure of **IIb** was confirmed by IR, ^1^H-NMR, mass spectrum and microanalysis. ^1^H-NMR showed the additional peak of deshielded CH_2_ protons at δ 4.97 ppm, while those of the ring were found at δ 3.85 ppm. Mass spectrum showed the distinctive isotopic peaks at *m/z* 211 and 209 in a 3:1 ratio due to the chlorine atom. The nucleophilic displacement of the chlorine atom with different secondary amines was done using different solvents, mainly ethanol, acetonitrile and ethyl acetate producing compounds **IIIa**–**j** and **VIa**–**e**. The structure of the produced derivatives was confirmed by IR, ^1^H-NMR, ^13^C-NMR, mass spectra and finally microanalyses. The acetyl protons were found slightly deshielded in the ^1^H-NMR at values δ 2.8–3.8 ppm. The specific piperazine pattern at δ 2.5 and 3.5 ppm in a 1:1 ratio in ^1^H-NMR and at δ 48 and 52 ppm in the ^13^C-NMR confirmed the products’ final structures. 

### 2.2. Pharmacology

**Figure 2:**
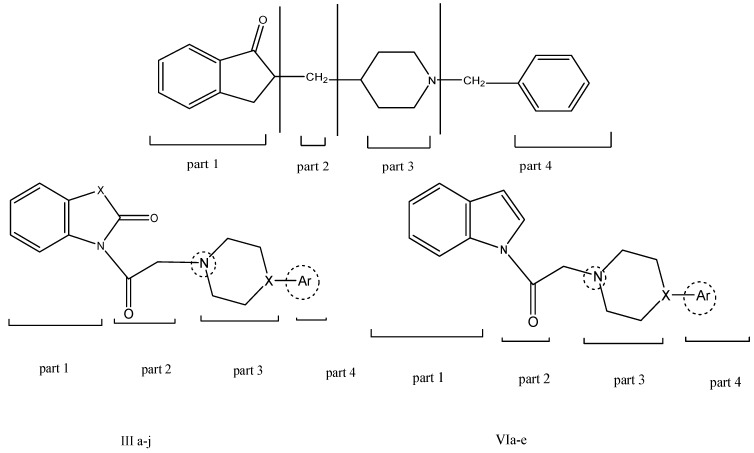
Resemblances between donepezil and synthesized derivatives, showing the four main parts important for biological activity.

Donepezil, which is a benzylpiperidine derivative, was chosen as a reference standard drug as it showed potent acetylcholinesterase inhibitor activity; it is thought to mimic the binding mode of ACh by structural similarity and therefore, be a competitive inhibitor of AChE [12] showing four main parts essential for its activity, those parts are the indanone moiety, a spacer, positive charge centre and a phenyl moiety [13] (Figure 2).

**Figure 3:**
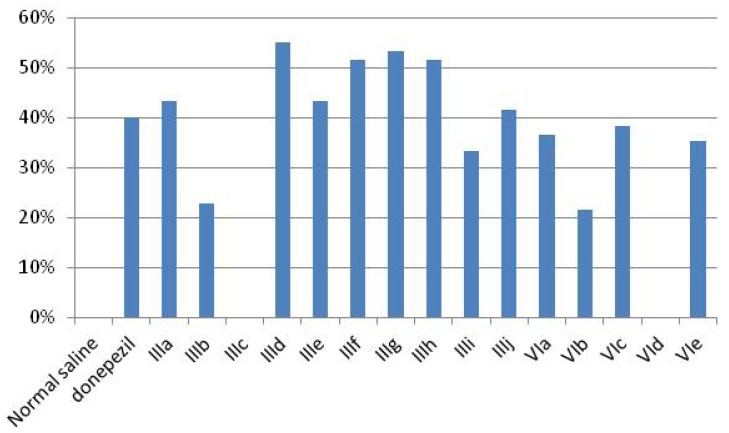
Bar chart representation of acetylcholinesterase percentage inhibition of normal saline, donepezil and the newly synthesized compounds.

On the previous basis, Ellman’s assay method [14] was performed on all of the newly synthesized compounds and on donepezil as standard to measure their inhibitory activity against acetylcholinesterase enzyme. Some of them such as **IIIa**, **IIId**, **IIIe**, **IIIf**, **IIIg**, **IIIh** and **IIIj** displayed better inhibitory activity than donepezil, Table 1, Figure 3.

**Table 1:** % Inhibition of AChE activity of donepezil and the synthesized new compounds.

Compound number	Choline Esterase content (U/ gm wet weight)	% inhibition
Normal saline	2815.20 ± 171.33	0%
donepezil	1689.20 ± 172.42	**40%**
IIIa	1595.28 ± 46.92	**43.33%**
IIIb	2170.56 ± 241.82	22.90%
IIIc	2873.70 ± 112.44	0%
IIId	1266.84 ± 119.62	**55%**
IIIe	1595.28 ± 136.79	43.33%
IIIf	1360.68 ± 136.79	**51.67%**
IIIg	1313.76 ± 93.84	**53.33%**
IIIh	1360.68 ± 114.93	**51.67%**
IIIi	1876.80 ± 74.187	33.33%
IIIj	1642.20 ± 128.50	**41.66%**
VIa	1785.84 ± 120.22	36.56%
VIb	2208.48 ± 190.22	21.55%
VIc	1736.04 ± 175.56	38.33%
VId	2955.96 ± 175.65	0%
VIe	1818.15 ± 112.31	35.42%

### 2.3. Molecular Modeling and Docking

Compounds **III_a_**, **III_d_** and **III_j_** showed good energy scores in addition to their superimposition with donepezil in the active site of AChE. These derivatives formed additional hydrogen bond interaction with Phe 288 at the AChE active site showing better fitting to the receptor which revealed that these derivatives could have similar pharmacological activity or even better activity than donepezil.

## 3. Experimental

### 3.1. Chemistry

All melting points were determined on a Stuart apparatus and the values given are uncorrected. IR spectra were determined on a Shimadzu IR 435 spectrophotometer at the Faculty of Pharmacy, Cairo University, Egypt. ^1^H-NMR and ^13^C-NMR spectra were recorded on Varian Gemini 200 MHz and 300 MHz spectrophotometer using TMS as internal standard. Chemical shift values are recorded in ppm on δ scale (Microanalysis Center, Cairo University, Egypt). Mass spectra were recorded on a Hewlett Packard 5988 spectrometer (Microanalysis Center, Cairo University) and a UPLC acquity TQ at the Faculty of Pharmacy, Cairo University, Egypt. Elemental analyses were carried out at the Microanalysis Center, Cairo University, Egypt; found values were within ±0.35% of the theoretical ones. Progress of the reactions was monitored using TLC sheets precoated with UV fluorescent silica gel Merck 60F 254 and were visualized using UV lamp. The synthesis of the target compounds is outlined in Scheme **1** and Scheme **2**. Compounds **Ia**, **IIa** and **V** were synthesized according to reported procedures [10,11]. 

#### 3.1.1. *1-(2-Chloroacetyl)indolin-2-one* (**IIb**)

A solution of **Ib** (9.044 g., 0.068 mol) in chloroacetyl chloride (100 g, 0.89 mol) was heated under reflux for 5 h. The reaction mixture was cooled and then ether (20 mL) was added. The precipitate formed was filtered, washed with (2 × 20 mL portions) ether, dried and crystallized from ethanol. M.p. (°C): 119–120, yield 55%, IR (KBr, cm^−1^): 3030.2970 (CH aromatic), 2927 (CH aliphatic), 1755, 1716 (C=O). ^1^H-NMR (DMSO-d_6_) δ: 3.85 (s, 2H, CH_2_ oxindole ring), 4.97 (s, 2H, CO–CH_2_–Cl), 7.22–8.09 (m, 4H, aromatic H) ppm. MS: *m/z* (% abundance) 209 (M^+^, 98.62%), 211 (M^+^+2, 35.41%). Analysis Calcd. C 57.29, H 3.85, N 6.68. Found C 57.33, H 3.83, N 6.64.

#### 3.1.2. General Procedure for the Synthesis of **IIIa**–**j**

To a solution of **IIa** or **IIb** (0.01 mol) in an appropriate solvent (20 mL), the appropriate secondary amine (0.02 mol) was added and the mixture was heated under reflux for 4–12 h. The reaction was cooled and poured into ice-cold water (25 mL). The product was extracted with chloroform. The chloroform layer was dried over anhydrous sodium sulphate and filtered. The filtrate was evaporated under reduced pressure to an oily product. Ether (25 mL) was added with stirring to the oily product and the separated solid was filtered, dried and crystallized from an appropriate solvent.

*1-(2-(4-Benzylpiperazin-1-yl)acetyl)indoline-2*,*3-dione* (**IIIa**). M.p. (°C): 90–92 (acetonitrile), yield 50%, IR (KBr, cm^−1^): 3050 (CH aromatic), 2920 (CH aliphatic), 1697, 1647 (C=O). ^1^H-NMR (DMSO-*d_6_*) δ: 2.56 (m, 4H, 2 × CH_2_ piperazine), 3.05 (m, 4H, 2 × CH_2_ piperazine), 3.55 (s, 4H, CO–CH_2_–N+CH_2_ benzyl), 7.23–8.49 (m, 9H, aromatic H) ppm. MS: *m/z* (% abundance) 363 (M^+^, 7.32%). Analysis Calcd. C 69.41, H 5.82, N 11.56. Found C 69.46, H 5.82, N 11.56.

*1-(2-(4-Phenylpiperazin-1-yl)acetyl)indoline-2*,*3-dione* **IIIb**. M.p. (°C): 136–138 (ethanol), yield 70%, IR (KBr, cm^−1^): 3155–3062 (CH aromatic), 2916 (CH aliphatic), 1689, 1651(C=O). ^1^H-NMR (DMSO-*d_6_*) δ: 3.06–3.07 (m, 4H, 2 × CH_2_ piperazine), 3.42–3.43 (m, 4H, CH_2_ piperazine), 3.7 (s, 2H, CO–CH_2_–N), 6.742–8.8 (4m, 9H, aromatic H) ppm. ^13^C-NMR (DMSO) δ: 48.51 (2×CH_2_ piperazine), 52.81 (2×CH_2_ piperazine), 61.82 (CO–CH_2_–N), 115.24–150.82 (aromatic C), 163.96 (C=O), 170.22 (C=O), 194.53 (C=O) ppm. MS: *m/z* (% abundance) 349 (M^+^, 5.1%). Analysis Calcd. C 68.75, H 5.48, N 12.03. Found C 68.81, H 5.51, N 12.06.

*1-(2-(4-(4-Methoxyphenyl)piperazin-1-yl)acetyl)indoline-2*,*3-*dione (**IIIc**). M.p. (°C): 172–174 (ethanol), yield 65%, IR (KBr, cm^−1^): 3000 (CH aromaic), 2920 (CH aliphatic), 1701, 1651 (C=O). ^1^H-NMR (DMSO-*d_6_*) δ: 2.69–2.73 (m, 4H, 2 × CH_2_ piperazine), 3.15–3.18 (m, 4H, 2 × CH_2_ piperazine), 3.27 (s, 2H, CO–CH_2_–N), 3.68 (s, 3H, OCH_3_), 6.76–8.83 (m, 8H, aromatic H) ppm. MS: *m/z* (% abundance) 379 (M^+^, 52.63%). Analysis Calcd. C 66.48, H 5.58, N 11.08. Found C 66.52, H 5.56, N 11.11.

*1-(2-(4-(2-Fluorobenzyl)piperazin-1-yl)acetyl)indoline-2*,*3-dione* (**IIId)**. M.p. (°C): 130–132 (ethanol), yield 52%, IR (KBr, cm^−1^): 3020 (CH aromatic), 2916 (CH aliphatic), 1681, 1647 (C=O). ^1^H-NMR (DMSO-*d_6_*) δ: 2.59 (m, 4H, 2 × CH_2_ piperazine), 3.05 (m, 4H, 2 × CH_2_ piperazine), 3.59 (s, 2H, CO–CH_2_–N), 3.75 (s, 2H, CH_2_ benzyl), 7.12–8.49 (m, 8H, aromatic H) ppm. MS: *m/z* (% abundance) 383 (M+2H, 0.09%). Analysis Calcd. C 66.13, H 5.29, N 11.02. Found C 66.19, H 5.56, N 11.10.

*1-(2-(4-Benzylpiperidin-1-yl)acetyl)indoline-2*,*3-dione* (**IIIe**). M.p. (°C): 100–102 (acetonitrile), yield 55%, IR (KBr, cm^−1^): 3078, 3020 (CH aromatic), 2916 (CH aliphatic), 1740, 1678, 1660 (C=O). ^1^H-NMR (DMSO-*d_6_*) δ: 1.49 (m, 5H, 2 × CH_2_ + CH piperidine), 2.44–2.76 (m, 4H, 2 × CH_2_ piperidine), 3.07 (s, 2H, CH_2_ benzyl), 3.83 (s, 2H, CO–CH_2_–N), 7.13–8.59 (m, 9H, aromatic H) ppm. MS: *m/z* (% abundance) 362 (M^+^, 1.91%). Analysis Calcd. C 72.91, H 6.12, N 7.73. Found C 72.98, H 6.15, N 7.75.

*1-(2-(4-Benzylpiperazin-1-yl)acetyl)indolin-2-one* (**IIIf**). M.p. (°C): 140–142 (ethanol), yield 52%, IR (KBr, cm^−1^): 3010, 3030 (CH aromatic), 2927 (CH aliphatic), 1675, 1635 (C=O). ^1^H-NMR (CDCl_3_) δ: 2.81 (m, 6H, 2 × CH_2_ piperazine + COCH_2_N), 3.25 (m, 6H, 2 × CH_2_ piperazine + CH_2_ oxindole ring), 3.61 (s, 2H, CH_2_ benzyl), 7.27–7.33 (m, 9H, aromatic H) ppm. MS: *m/z* (% abundance) 350(M^+^+H) (0.05). Analysis Calcd. C 72.18, H 6.635, N 12.03. Found C 72.36, H 6.91, N 11.89.

*1-(2-(4-Phenylpiperazin-1-yl)acetyl)indolin-2-one* (**IIIg**). M.p. (°C): 178–180 (acetonitrile), yield 51%, IR (KBr, cm^−1^): 3059 (CH aromatic), 2930 (CH aliphatic), 1708, 1675 (C=O). ^1^H-NMR (CDCl_3_) δ: 1.57 (m, 4H, 2 × CH_2_ piperazine), 2.99 (m, 4H, 2 × CH_2_ piperazine), 3.7 (s, 2H, COCH_2_N), 3.82 (s, 2H, CH_2_ oxindole ring), 6.91–7.27 (m, 9H, aromatic H) ppm. MS: *m/z* (% abundance) 335 (M^+^, 2.11%). Analysis Calcd. C 71.62, H 6.31, N 12.52. Found C 71.68, H 6.36, N 12.59.

*1-(2-(4-(4-Methoxyphenyl)piperazin-1-yl)acetyl)indolin-2-one* (**IIIh**). M.p. (°C): 138–140 (acetonitrile), yield 50%, IR (KBr, cm^−1^): 3059, 3040 (CH aromatic), 2920 (CH aliphatic), 1643 (C=O). ^1^H-NMR (CDCl_3_) δ: 1.57 (m, 4H, 2 × CH_2_ piperazine), 2.99–3.8 (m, 8H, 2 × CH_2_ piperazine+COCH_2_N+CH_2_ oxindole ring), 6.92–7.26 (m, 8H, aromatic H) ppm. MS: *m/z* (% abundance) 365 (M^+^, 19.41%). Analysis Calcd. C 69.02, H 6.34, N 11.50. Found C 69.11, H 6.32, N 11.81.

*1-(2-(4-(2-Fluorobenzyl)piperazin-1-yl)acetyl)indolin-2-one* (**IIIi**). M.p. (°C): 90–92 (ethanol), yield 45%, IR (KBr, cm^−1^): 3059, 3040 (CH aromatic), 2924 (CH aliphatic), 1701, 1689 (C=O).^1^H-NMR (DMSO-*d_6_*) δ: 2.5 (m, 4H, 2 × CH_2_ piperazine), 3.3 (m, 6H, 2 × CH_2_ piperazine + COCH_2_N), 3.45 (m, 4H, CH_2_ benzyl+CH_2_ oxindole ring), 6.79–7.2 (m, 8H, aromatic H) ppm. MS: *m/z* (% abundance) 368 (M^+^+H, 8.19%). Analysis Calcd. C 68.65, H 6.04, N 11.44. Found C 68.71, H 6.12, N 11.49.

*1-(2-(4-Benzylpiperidin-1-yl)acetyl)indolin-2-one* (**IIIj**). M.p. (°C): 98–100 (ethanol), yield 50%, IR (KBr, cm^−1^): 3078, 3032 (CH aromatic), 2900 (CH aliphatic), 1701, 1660 (C=O).^1^H-NMR (DMSO-*d_6_*) δ: 1.90 (m, 5H, 2 × CH_2_ + CH piperidine), 3.31 (m, 6H, 2 × CH_2_ piperidine + CH_2_ benzyl) 3.45 (m, 4H, COCH_2_N + CH_2_ oxindole ring), 6.79–7.21 (m, 9H, aromatic H) ppm. MS: *m/z* (% abundance) 348 (M^+^, 0.59%). Analysis Calcd. C 78.83, H 6.94, N 8.04. Found C 75.88, H 6.96, N 8.03.

#### 3.1.3. General Procedure for the Synthesis of **VIa**–**e**

To a solution of **V** (0.01 mol) in dry benzene (20 mL), the appropriate amine (0.01 mol) and triethylamine (5 drops) were added. The reaction mixture was heated under reflux for 5–7 h. The reaction mixture was cooled and the separated solid was filtered, dried and crystallized from the appropriate solvent.

*2-(4-Benzylpiperazin-1-yl)-1-(1H-indol-1-yl)ethanone* (**VIa**). M.p. (°C): 158–160 (acetonitrile), yield 60%, IR (KBr, cm^−1^): 3030 (CH aromatic), 2927, 2912 (CH aliphatic), 1654 (C=O). ^1^H-NMR (DMSO-*d_6_*) δ: 2.49 (m, 4H, 2 × CH_2_ piperazine), 3.03 (m, 4H, 2 × CH_2_ piperazine), 3.30 (s, 2H, CO–CH_2_–N), 4.0 (s, 2H, CH_2_ benzyl), 6.73–8.34 (m, 11H, aromatic H) ppm. MS: *m/z* (% abundance) 333 (M^+^, 14.65%). Analysis Calcd. C 75.64, H 6.95, N 12.60. Found C 75.68, H 6.91, N 12.63.

*1-(1H-Indol-1-yl)-2-(4-phenylpiperazin-1-yl)ethanone* (**VIb**). M.p. (°C): 223–225 (ethyl acetate), yield 65%, IR (KBr, cm^−1^): 3100 (CH aromatic), 2924 (CH aliphatic), 1658 (C=O). ^1^H-NMR (DMSO-*d_6_*) δ: 3.17 (m, 4H, 2 × CH_2_ piperazine), 3.32 (m, 4H, 2 × CH_2_ piperazine), 3.38 (s, 2H, CO–CH_2_–N), 6.83–7.28 (m, 11H, aromatic H) ppm. MS: *m/z* (% abundance) 319 (M^+^, 0.28%). Analysis Calcd. C 75.21, H 6.63, N 13.18. Found C 75.19, H 6.60, N 13.18.

*1-(1H-Indol-1-yl)-2-(4-(4-methoxyphenyl)piperazin-1-yl)ethanone* (**VIc**). M.p. (°C): 183–185 (acetonitrile), yield 60%, IR (KBr, cm^−1^): 3150 (CH aromatic), 2935 (CH aliphatic), 1712 (C=O). ^1^H-NMR (DMSO-*d_6_*) δ: 2.73 (m, 4H, 2 × CH_2_ piperazine), 3.01 (m, 4H, 2 × CH_2_ piperazine), 3.70 (s, 2H, CO–CH_2_–N), 3.88 (s, 3H, OCH_3_), 6.82–8.36 (m, 10H, aromatic H) ppm. ^13^C-NMR (DMSO) δ: 49.48 (2 × CH_2_ piperazine), 52.31 (2 × CH_2_ piperazine), 55.05(CO–CH_2_–N), 60.87 (OCH_3_), 107.96–152.83 (aromatic C), 168.56 (C=O) ppm. MS: *m/z* (% abundance) 349 (M^+^, 0.03%). Analysis Calcd. C 72.18, H 6.63, N 12.03. Found C 72.18, H 6.63, N 12.11.

*2-(4-(2-Fluorobenzyl)piperazin-1-yl)-1-(1H-indol-1-yl)ethanone* (**VId**). M.p. (°C): 136–138 (acetonitrile), yield 62%, IR (KBr, cm^−1^): 3030 (CH aromatic), 2916 (CH aliphatic), 1650 (C=O). ^1^H-NMR (DMSO-*d_6_*) δ: 2.50 (m, 4H, 2 × CH_2_ piperazine), 3.05 (m, 4H, 2 × CH_2_ piperazine), 3.30 (s, 2H, CO–CH_2_–N), 4.01 (s, 2H, CH_2_ benzyl), 6.75–8.34 (m, 10H, aromatic H) ppm. MS: *m/z* (% abundance) 351 (M^+^, 3.87%), 86 (C_4_H_6_NO, 100%). Analysis Calcd. C 71.77, H 6.31, N 11.96. Found C 71.72, H 6.34, N 11.90. 

*2-(4-Benzylpiperidin-1-yl)-1-(1H-indol-1-yl)ethanone* (**VIe**). M.p. (°C): 204–206 (acetonitrile), yield 70%, IR (KBr, cm^−1^): 3055, 3024 (CH aromatic), 2924, 2850 (CH aliphatic), 1658 (C=O). ^1^H-NMR (DMSO-*d_6_*) δ: 1.69 (m, 5H, 2 × CH_2_ + CH piperidine), 3.06 (m, 4H, 2 × CH_2_ piperidine), 3.53 (s, 2H, CH_2_ benzyl), 4.9 (s, 2H, CO–CH_2_–N), 6.85–8.35 (m, 11H, aromatic H) ppm. MS: *m/z* (% abundance) 332 (M^+^, 1.1%). Analysis Calcd. C 79.48, H 7.28, N 8.43. Found C 79.51, H 7.28, N 8.45.

### 3.2. Pharmacology

Adult male albino Wister rats weighing 180–200 g were used in the present study. Rats were purchased from the animal house of El-Nile Company (Cairo, Egypt). Rats were kept under constant laboratory conditions and were allowed free access to food and water throughout the period of investigation. The tested compounds were orally administered at concentration of 2.6351 mM concentration [equivalent to that of donepezil], the compounds were mixed with Tween 80, diluted with distilled water and administered orally. After 30 min rats were killed, decapitated, then brains were carefully removed and homogenized in normal saline (pH 7.4).

Inhibitory activity against AChE was evaluated at 37 °C by the colorimetric method reported by Ellman *et al*. [14]. The final concentration containing test compound of the assay solution consisted of 0.1 Mol sodium phosphate buffer (pH 8.0), 0.3 mM 5,5-dithiobis-2-nitrobenzoic acid (DTNB, Ellman’s reagent) and 0.5 mM acetylthiocholine iodide as substrate of the enzymatic reaction. The principle of the assay is based on that the thio-ester substrate acetylthiocholine (AchSC) is hydrolyzed by the enzyme, releasing a sulfhydrylic group able to react with bis(3-carboxy-4-nitrophenyl) disulfide (Ellman’s reagent). The kinetics of this activity is then followed with the use of a spectrophotometer at 412 nm for 2 min. Absorbance is measured at 0, 1 and 2 min and the mean change in absorbance (ΔA) is calculated for each sample the values were recorded. The AChE inhibition was determined for each compound. Each assay was run in triplicate and each reaction was repeated three independent times.

### 3.3. Molecular Modeling and Docking

Docking was carried out on an Intel Pentium 1.6 GHz processor, 512 MB memory with Windows XP operating system using Molecular Operating Environment (MOE 2008.10; Chemical Computing Group, Montreal, Canada) as the computational software.

The 3D structure of the acetylcholine esterase complexed with donepezil was obtained from the Protein Data Bank (PDB ID: 1EVE) at Research Collaboration for Structural Bioinformatics (RCSB) protein data bank base [15] with a 2.5 Å resolution. The compounds were constructed using the builder module and were energy minimized using the MMFF94x force field. Hydrogen and partial charges were added to the system using Protonate 3D application.

In the present work, all the prepared new compounds were docked using a rigid receptor/fexible ligand approach adopting five energy maps which are hydrophobicity, electrostatic, hydrogen bond formation and two Van der Waal parameters. The docking scores were expressed in negative energy terms; the lower the binding free energy, the better the binding affinity.

**Figure 4:**
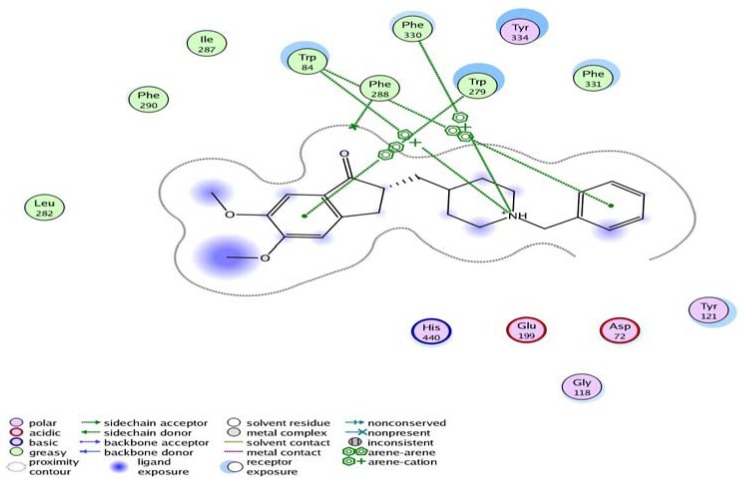
Interactions of donepezil with the amino acids of the AChE active site.

**Figure 5:**
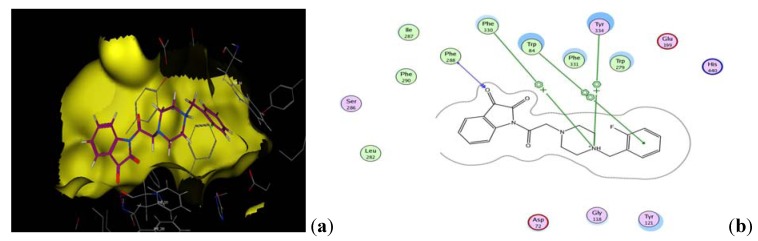
(**a**) Docked pose of **III_d_** in the AChE binding site generated by MOE docking. (**b**) Simplified structure showing interaction between **VII_c_** and the aromatic residues in the AChE active site.

The docking study displayed that most of the designed compounds showed promising affinity to inhibit acetylcholinesterase. The data obtained from docking of the target compounds were explained showing amino acids interactions and hydrogen bond length. Table 2, Figure 4 and Figure 5.

**Table 2:** MOE Scores of Donepezil, compounds **III_a_**_–**j**_, **Vi_a_**_–**e**_ and bonds formed with amino acid residues and their lengths.

Compound number	Type of interaction (Amino acid residues, length of bond in A°)	Binding Energy Score (Kcal/mol) *
Donepezil	π-π (Trp279), π-π, π-cation (Trp84), π-cation (Phe330)	−31.1758
III_a_	π-π (Trp84), π-π (Trp279), π-cation (Tyr334), H-bond (Tyr121, 2.93), H-bond (Tyr70, 3.04)	−28.4850
III_b_	H-bond (Phe288, 2.65), π-cation (His440)	−24.7083
III_c_	----------	−24.5012
**III_d_**	**π-π (Trp84), π-cation (Phe330), π-cation (Tyr334), H-bond (Phe238, 2.69)**	**−23.4711**
III_e_	π-π (Trp84), π-cation (Tyr334), H-bond (Phe288, 2.67)	−24.3158
III_f_	π-π (Trp279), π-π (Trp84), π-cation (Phe330), π-cation (Tyr334), H-bond (Phe288,2.56)	−27.1238
III_g_	π-π(Trp84), H-bond (Tyr121, 2.64)	−22.6958
III_h_	π-π (Trp279), π-π (Trp84), H-bond (Phe268,3.8)	−24.6397
III_i_	π-π (Trp279), π-π (Trp84), π-cation (Phe330)	−26.2485
III_j_	π-π (Trp279), π-π (Trp84), π-cation (Tyr334), H-bond (Phe288, 2.56)	−27.1238
VI_a_	π-π (Trp84), π-π(Trp279), π-cation (Phe330), π-cation (Tyr334)	−25.1652
VI_b_	π-π (Trp84)	−23.7943
VI_c_	π-π (Trp279), π-π (Trp84)	−20.0750
VI_d_	π-cation (Phe330), π-cation (Tyr334)	−26.2897
VI_e_	π-π (Trp84), π-cation (Phe330), π-cation (Tyr334), H-bond (Tyr121, 2.48)	−23.8138

## 4. Conclusions

Compound **IIId** showed inhibitory activity better than donepezil owing mainly to its additional 3-oxo group and 2-flourobenzyl substitution, as well as retention of the acetamido spacer in addition to its extra binding to the receptor by the H-bond with Phe288. Compounds **IIIf**, **IIIg** and **IIIh** showed inhibitory activity close to **IIId**, and still better activity than donepezil. Compounds **IIIa**, **IIIe** and **IIIj** showed moderate activity but still retained similar inhibitory activity to donepezil owing to the better fitting to the receptor with the extra H-bond. The highest activity was observed amongst isatin and oxindole derivatives, which proves that the 2-one group increases activity. The acetamido spacer was crucial for activity [2,13]. Moreover, the benzyl group showed better activity than phenyl group and better fitting to the receptor. From the previous results, the extra binding to the receptor with the H-bond lead to better pharmacological activity. 

## Supplementary Materials

Supplementary materials can be accessed at: http://www.mdpi.com/1420-3049/17/5/4811/s1.
