# Supplementary figures and images for: Synthesis of New Indole Derivatives Structurally Related to Donepezil and Their Biological Evaluation as Acetylcholinesterase Inhibitors
Source: Molecules. 2012 Apr 25;17(5):4811–23. doi: 10.3390/molecules17054811 (PMC6268345; doi:10.3390/molecules17054811)

# Supplementary Materials

<sup>1</sup>H-NMR of compound IIIe.

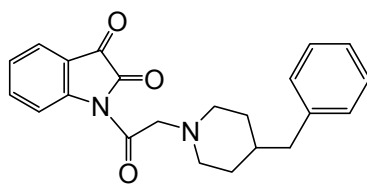

IIIe

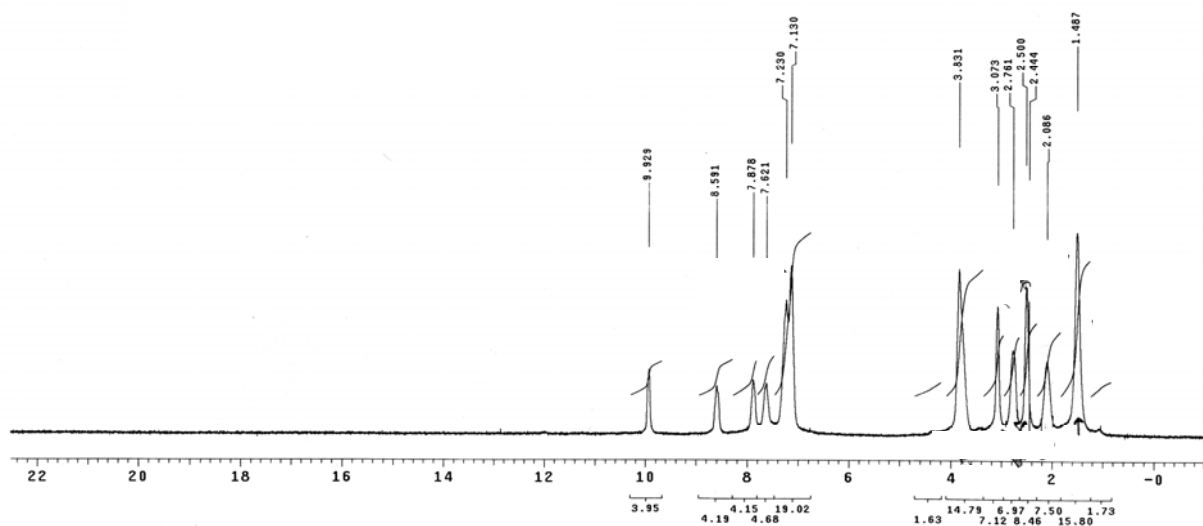

Supplement: Supplementary file 1 [file molecules-17-04811-s001.pdf]
